# Linking gene regulation and the exo-metabolome: A comparative transcriptomics approach to identify genes that impact on the production of volatile aroma compounds in yeast

**DOI:** 10.1186/1471-2164-9-530

**Published:** 2008-11-07

**Authors:** Debra Rossouw, Tormod Næs, Florian F Bauer

**Affiliations:** 1 Institute for Wine Biotechnology, University of Stellenbosch, Stellenbosch, South Africa; 2 Centre for Biospectroscopy and Data Modelling, NOFIMA FOOD, Matforsk AS, Oslovegen 1, 1430 Ås, Norway

## Abstract

**Background:**

'Omics' tools provide novel opportunities for system-wide analysis of complex cellular functions. Secondary metabolism is an example of a complex network of biochemical pathways, which, although well mapped from a biochemical point of view, is not well understood with regards to its physiological roles and genetic and biochemical regulation. Many of the metabolites produced by this network such as higher alcohols and esters are significant aroma impact compounds in fermentation products, and different yeast strains are known to produce highly divergent aroma profiles. Here, we investigated whether we can predict the impact of specific genes of known or unknown function on this metabolic network by combining whole transcriptome and partial exo-metabolome analysis.

**Results:**

For this purpose, the gene expression levels of five different industrial wine yeast strains that produce divergent aroma profiles were established at three different time points of alcoholic fermentation in synthetic wine must. A matrix of gene expression data was generated and integrated with the concentrations of volatile aroma compounds measured at the same time points. This relatively unbiased approach to the study of volatile aroma compounds enabled us to identify candidate genes for aroma profile modification. Five of these genes, namely *YMR210W*, *BAT1*, *AAD10*, *AAD14 *and *ACS1 *were selected for overexpression in commercial wine yeast, VIN13. Analysis of the data show a statistically significant correlation between the changes in the exo-metabome of the overexpressing strains and the changes that were predicted based on the unbiased alignment of transcriptomic and exo-metabolomic data.

**Conclusion:**

The data suggest that a comparative transcriptomics and metabolomics approach can be used to identify the metabolic impacts of the expression of individual genes in complex systems, and the amenability of transcriptomic data to direct applications of biotechnological relevance.

## Background

Commercial wine yeast strains have been selected to meet specific requirements of wine producers with regard to phenotypical traits such as fermentation performance, general stress resistance, the profile of aromatic compounds produced, the ability to release enzymes or mannoproteins of oenological relevance and many more [1]. As a result, more than 200 different yeast strains, almost exclusively of the species *Saccharomyces cerevisiae *are currently produced and sold in the global industry. Many research and development programs have focused on improving specific aspects of wine yeast strains [1]. However, many of the relevant traits are of a polygenic nature, and our understanding of the genetic and molecular regulation of complex, commercially relevant phenotypes is limited [2]. In this paper, we investigate the possibility of using a holistic systems biology approach to identify genes that impact on volatile aroma compound production during fermentation. The approach is based on combining comparative transcriptomics and aroma metabolomics of five commercial wine yeast strains that produce significantly different aroma profiles.

During alcoholic fermentation, *Saccharomyces cerevisiae *strains convert sugars to ethanol, but also produce a large number of volatile aroma compounds, including fatty acids, higher alcohols and esters (table 1). Many of these compounds are important flavor and aroma compounds in wine and beer, and different strains of *S. cerevisiae *are well known to impart significantly different aroma profiles to the final product.

**Table 1:** Exo-metabolites measured in this study

Primary metabolites	Organic acids	Higher alcohols	Esters	Acids	Fatty Acids
Glucose	Citrate	Methanol	Ethyl Acetate	Valeric Acid	Octanoic Acid
Fructose	Malate	Propanol	Hexyl acetate	Propionic Acid	Decanoic Acid
Glycerol	Acetate	Isoamyl alcohol	Isoamyl Acetate	Iso-Valeric Acid	Ethyl Caprylate
Ethanol		2-Phenyl Ethanol	2-Phenylethyl Acetate	Iso-Butyric Acid	Ethyl Lactate
		Isobutanol		Butyric Acid	Ethyl Caprate

		Butanol			

**Figure 1:**
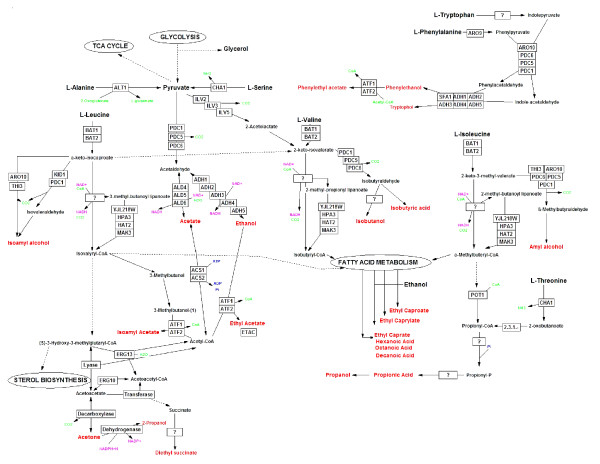
**Diagrammatic representation of pathways associated with aroma production and links to associated metabolic activities.** Dashed arrows are used when one or more intermediates or reactions are omitted. Red font is used to identify relevant aroma compounds. Full gene names and functions can be viewed in the appendix. The main pathway for the production of higher alcohols is known as the Erlich Pathway [3]: it involves three basic enzyme activities and starts with the deamination of leucine, valine and isoleucine to the corresponding α-ketoacids. Each α-ketoacid is subsequently decarboxylated and converted to its branched-chain aldehyde [4-6]. The final step is an alcohol dehydrogenase-catalyzed step which could potentially be catalyzed by the seven putative aryl alcohol dehydrogenase genes [7], and the seven alcohol dehydrogenase genes [8]. Finally ester formation involves the enzyme-catalyzed condensation reaction between a higher alcohol and an activated acyl-coenzyme A [9-11]. Fatty acids are derived from fatty acid biosynthesis, but can also be produced as intermediates of the higher alcohol and ester producing pathways [9].

The metabolic pathways responsible for the production of these compounds are responsive to many factors including the availability of precursors, different types of stress, the cellular redox potential and the energy status of the cell [3-11]. These pathways are not linear, but rather form a network of interlinked reactions converging and diverging from shared intermediates (figure 1). Moreover, intermediates are not only shared between the different 'branches' of aroma compound production, but also with other pathways related to fatty acid metabolism, glycolysis, stress tolerance and detoxification to name a few.

Most of the genes encoding the enzyme activities of the aroma network are also co-regulated by transcription factors that are related to total nitrogen and amino acid availability [12]. Thus the nutritional status of the cell as well as the nutrient composition of the growth media throughout fermentation plays a vital role in determining the aroma profile produced by the fermenting yeast. A further complication is due to the fact that very little is known about the kinetics of individual enzymes involved in these pathways. What is clear is that a number of these enzymes are capable of catalyzing both the forward and reverse reactions, depending on the ratios of substrates to end products, as well as the prevailing redox balance of the cell [13-15]. The various dehydrogenase- catalyzed reactions which are integral to most branches of aroma production are particularly sensitive to the ratios of enzyme co-factors such as NAD and NADH, with obvious ramifications regarding the directionality of various key reactions [16]. This intricate lattice of chemical and biological interactions makes interpretation of individual gene and enzyme contributions problematic in the context of aroma compound production as a whole (figure 1). Indeed, individual parts of the system can combine and interact in unexpected ways, giving rise to emergent properties or functions that would not be anticipated by studying a single part of the system. Such systems are thus irreducible, and cannot be understood by dissection and analysis of a single part at a time. In recognition of the complex and intricate nature of this process we have sought to follow an 'omic' approach in the study of aroma compound production.

In the present study our goal was to compare the aroma-relevant exo-metabolomes of five industrial yeast strains at three different stages of fermentation, and to align these data with gene expression data obtained through microarray-based genome-wide transcription analysis. This enabled the incorporation of gene expression levels and aroma compound production into multivariate statistical models. By using these models as a predictive tool various genes were identified as potential candidates for overexpression in order to increase/decrease the levels of key aroma compounds during fermentation. To verify whether genes whose differential regulation appeared most strongly linked to the differences observed in the aroma profiles of different strains were indeed impacting on aroma compound metabolism, five of these genes were individually overexpressed in one of the industrial strains. The data indicate that these genes indeed impacted significantly on the aroma profiles produced by the modified strains. Moreover, the pattern of changes observed was significantly correlated to the pattern predicted through the comparative analysis of transcriptome and metabolome. The data therefore clearly support our hypothesis that direct comparative analysis of transcriptomes and metabolomes can be used for the identification of genes that affect specific metabolic networks and for predicting the impact of the expression of such genes on these networks.

## Results

### Fermentation kinetics and metabolite formation

**Figure 2:**
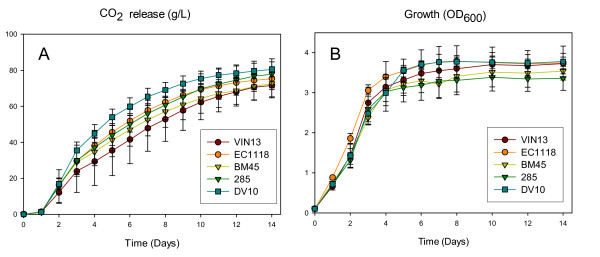
**Growth rate (frame A) and CO_2 _release (frame B) of the five commercial wine yeast strains during alcoholic fermentation.** Values are the average of 4 biological repeats ± standard deviation.

**Figure 3:**
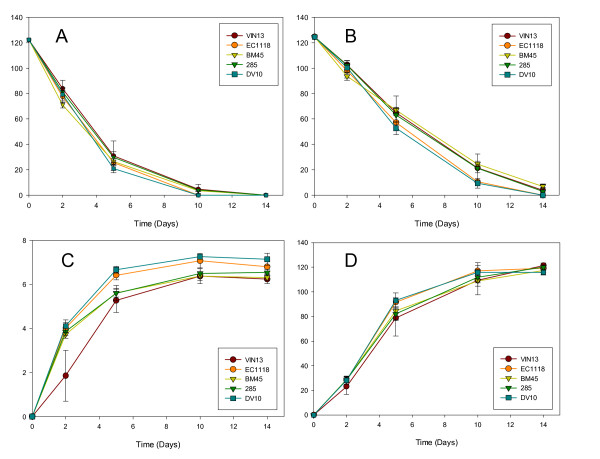
**Fermentation kinetics of the five yeast strains used in this study: Glucose utilization (A), fructose utilization (B), glycerol production (C) and ethanol production (D).** All y-axis values are in g.l^-1 ^and refer to extracellular metabolite concentrations in the synthetic must. Values are the average of 4 biological repeats ± standard deviation.

Fermentation behaviour of all five strains in our conditions followed typical wine fermentation patterns. All five strains fermented the synthetic must to dryness within the monitored period, broadly followed similar growth patterns (figure 2) and showed similar rates of fructose and glucose utilization as well as ethanol and glycerol production (figure 3). This is to be expected, as all five strains are widely used in the wine industry and are optimized for fermentation performance.

On the other hand, the strains did show significant variability regarding the volatile organoleptic compounds produced during fermentation (tables 2, 3, 4), suggesting that these 'secondary' pathways of higher alcohol and ester production are less conserved between different strains.

**Table 2:** Volatile alcohols and esters present in the fermentation media at day 2 of fermentation

Day2	Threshold	Odor	VIN13	EC1118	BM45	285	DV10
Ethyl Acetate	12	Apple, Pineapple, fruity, solvent, balsamic	4.87 ± 1.22	7.38 ± 0.72	7.32 ± 1.07	5.43 ± 1.27	7.32 ± 1.94
Methanol			0 ± 0.00	0 ± 0.00	0 ± 0.00	42.59 ± 3.08	41.72 ± 4.08
Propanol	306	Alcohol, ripe fruit, stupefying	27.16 ± 11.35	21.04 ± 4.26	20.87 ± 2.08	19.24 ± 2.60	23.15 ± 6.42
Isobutanol	74	Alcohol, nail polish	5.7 ± 1.25	6.77 ± 0.63	8.3 ± 0.81	7.28 ± 0.40	7.04 ± 0.61
Isoamyl Acetate	60	Solvent marzipan, malt	bd	bd	bd	bd	0.05 ± 0.01
Butanol	50	Medicinal, wine-like	bd	bd	bd	bd	bd
Isoamyl alcohol			26.54 ± 5.51	31.23 ± 2.74	30.39 ± 3.12	27.31 ± 1.87	31.09 ± 4.74
Hexyl acetate	0.67	Apple, cherry, pear, floral	bd	bd	bd	bd	0.01 ± 0.01
Ethyl Lactate	150	Fruity, buttery	bd	bd	4.08 ± 0.07	bd	bd
Ethyl Caprylate	0.58	sweet pear banana	bd	bd	bd	bd	0.05 ± 0.03
Acetic Acid		Vinegar	549 ± 56	766 ± 19	841 ± 22	756 ± 32	733 ± 60
Propionic Acid			1.59 ± 0.62	1.55 ± 0.11	1.78 ± 0.32	1.7 ± 0.35	1.74 ± 0.15
Iso-Butyric Acid	8.1	Rancid butter cheese	0.75 ± 0.11	0.6 ± 0.03	0.62 ± 0.05	0.56 ± 0.07	0.7 ± 0.04
Butyric Acid	2.2	Rancid cheese, sweet	1.10 ± 0.27	1.19 ± 0.06	1.03 ± 0.06	0.96 ± 0.09	1.23 ± 0.08
Ethyl Caprate	0.5	Brandy fruity grape floral	0.02 ± 0.01	0.07 ± 0.03	0.09 ± 0.00	0.09 ± 0.01	0.11 ± 0.01
Iso-Valeric Acid	0.7	Rancid cheese putrid	0.36 ± 0.08	0.33 ± 0.13	0.28 ± 0.04	0.27 ± 0.04	0.340.05
Diethyl Succinate	1.2	Fruity, melon	bd	bd	bd	bd	bd
Valeric Acid		SWEATY	0.22 ± 0.05	0.29 ± 0.04	0.26 ± 0.08	0.29 ± 0.06	0.29 ± 0.06
2-Phenylethyl Acetate	1.8	rosy honey fruity	0.22 ± 0.05	0.51 ± 0.03	0.32 ± 0.03	0.33 ± 0.03	0.57 ± 0.03
2-Phenyl Ethanol	200	Rose, honey	9.07 ± 2.37	12.8 ± 0.41	13.38 ± 1.07	14.88 ± 1.33	12.67 ± 1.36
Octanoic Acid	10	Fatty, rancid, oily, soapy faint fruity	0.81 ± 0.04	1.57 ± 0.05	1.01 ± 0.05	1.11 ± 0.07	1.74 ± 0.08
Decanoic Acid	6	Fatty, rancid	0.66 ± 0.07	1.46 ± 0.02	1.06 ± 0.12	1.22 ± 0.13	1.7 ± 0.11

* Values are expressed as mg.L^-1 ^and are the average of 4 biological repeats ± standard deviation. Metabolites present at concentrations below the detection limit are indicated by "bd".

**Table 3:** Volatile alcohols and esters present in the fermentation media at day 5 of fermentation

Day5	Threshold	Odor	VIN13	EC1118	BM45	285	DV10
Ethyl Acetate	12	Apple, Pineapple, fruity, solvent, balsamic	15.8 ± 4.43	16.923 ± 4.22	14.37 ± 1.49	14.54 ± 1.9	23.87 ± 2.21
Methanol			15.80 ± 4.49	36.13 ± 6.26	0 ± 0.00	29.89 ± 34.7	34.42 ± 16.84
Propanol	306	Alcohol, ripe fruit, stupefying	77.11 ± 20.23	55.58 ± 7.76	31.67 ± 6.38	35.19 ± 7.65	58.1 ± 5.38
Isobutanol	74	Alcohol, nail polish	8.96 ± 2.15	11.98 ± 0.63	13.57 ± 1.60	11.54 ± 0.85	11.66 ± 0.60
Isoamyl Acetate	60	Solvent marzipan, malt	0.11 ± 0.04	0.09 ± 0.01	0.07 ± 0.01	0.07 ± 0.01	0.10 ± 0.01
Butanol	50	Medicinal, wine-like	0.95 ± 0.11	0.89 ± 0.05	0.7 ± 0.06	0.77 ± 0.05	0.98 ± 0.02
Isoamyl alcohol			57.02 ± 10.92	61.46 ± 6.52	51.46 ± 5.98	49.32 ± 3.95	67.15 ± 8.09
Hexyl acetate	0.67	Apple, cherry, pear, floral	0.01 ± 0.02	0.16 ± 0.01	0.1 ± 0.01	0.04 ± 0.01	0.16 ± 0.01
Ethyl Lactate	150	Fruity, buttery	4.33 ± 0.14	4.24 ± 0.12	bd	bd	4.48 ± 0.06
Ethyl Caprylate	0.58	sweet pear banana	0.08 ± 0.02	0.11 ± 0.02	0.07 ± 0.01	0.07 ± 0.00	0.09 ± 0.01
Acetic Acid		Vinegar	1306.24 ± 74.24	879.5 ± 22.00	1000.58 ± 27.72	703.33 ± 43.78	1105.93 ± 24.80
Propionic Acid			2.18 ± 0.35	2.32 ± 0.28	2.15 ± 0.29	2.01 ± 0.22	2.52 ± 0.19
Iso-Butyric Acid	8.1	rancid butter cheese	0.58 ± 0.12	0.58 ± 0.04	0.59 ± 0.04	0.54 ± 0.04	0.76 ± 0.04
Butyric Acid	2.2	rancid cheese, sweet	1.97 ± 0.41	2.50 ± 0.15	1.53 ± 0.30	1.57 ± 0.31	2.77 ± 0.62
Ethyl Caprate	0.5	brandy fruity grape floral	0.25 ± 0.05	0.32 ± 0.03	0.18 ± 0.03	0.2 ± 0.02	0.33 ± 0.05
Iso-Valeric Acid	0.7	rancid cheese putrid	0.59 ± 0.11	0.62 ± 0.04	0.53 ± 0.08	0.54 ± 0.16	0.66 ± 0.05
Diethyl Succinate	1.2	Fruity, melon	bd	bd	bd	bd	bd
Valeric Acid		SWEATY	0.57 ± 0.25	0.38 ± 0.07	0.23 ± 0.02	0.26 ± 0.03	0.39 ± 0.03
2-Phenylethyl Acetate	1.8	rosy honey fruity	0.33 ± 0.03	0.63 ± 0.02	0.35 ± 0.02	0.32 ± 0.03	0.68 ± 0.05
2-Phenyl Ethanol	200	Rose, honey	12.71 ± 2.47	14.98 ± 0.58	16.35 ± 1.68	17.21 ± 0.83	16.33 ± 0.87
Octanoic Acid	10	Fatty, rancid, oily, soapy faint fruity	0.95 ± 0.2	1.43 ± 0.05	0.9 ± 0.08	0.88 ± 0.09	1.59 ± 0.09
Decanoic Acid	6	Fatty, rancid	1.5 ± 0.41	2.86 ± 0.38	1.32 ± 0.15	1.42 ± 0.13	3.01 ± 0.22

* Values are expressed as mg.L^-1 ^and are the average of 4 biological repeats ± standard deviation. Metabolites present at concentrations below the detection limit are indicated by "bd".

**Table 4:** Volatile alcohols and esters present in the fermentation media at day 14 of fermentation

Day14	Threshold	Odor	VIN13	EC1118	BM45	285	DV10
Ethyl Acetate	12	Apple, Pineapple, fruity, solvent, balsamic	29.56 ± 7.56	28.79 ± 1.83	23.58 ± 2.42	23.24 ± 1.87	27.83 ± 1.11
Methanol			68.39 ± 4.8	62.3 ± 20.12	70.87 ± 7.02	63.92 ± 4.49	62.18 ± 6.21
Propanol	306	Alcohol, ripe fruit, stupefying	83.23 ± 13.34	53.5 ± 7.49	39.72 ± 4.13	41.63 ± 5.337	64.14 ± 6.96
Isobutanol	74	Alcohol, nail polish	11.27 ± 2.80	15.44 ± 1.30	19.58 ± 2.03	15.18 ± 1.15	15.33 ± 0.76
Isoamyl Acetate	60	Solvent marzipan, malt	0.10 ± 0.03	0.09 ± 0.01	0.07 ± 0.01	0.06 ± 0.00	0.09 ± 0.01
Butanol	50	Medicinal, wine-like	1.36 ± 0.14	1.26 ± 0.08	1.02 ± 0.18	1.03 ± 0.05	1.32 ± 0.04
Isoamyl alcohol			67.29 ± 4.89	71.44 ± 2.54	68.17 ± 8.81	61.59 ± 4.25	76.86 ± 6.50
Hexyl acetate	0.67	Apple, cherry, pear, floral	0.05 ± 0.04	0.14 ± 0.01	0.1 ± 0.00	0.1 ± 0.00	0.14 ± 0.00
Ethyl Lactate	150	Fruity, buttery	3.86 ± 0.19	3.96 ± 0.04	bd	bd	3.92 ± 0.09
Ethyl Caprylate	0.58	sweet pear banana	0.07 ± 0.00	0.11 ± 0.01	0.07 ± 0.01	0.07 ± 0.01	0.13 ± 0.01
Acetic Acid		Vinegar	1616 ± 158	1061 ± 120	1197 ± 133	838 ± 114	1251 ± 71
Propionic Acid			1.96 ± 0.39	2.84 ± 0.22	2.18 ± 0.47	2.19 ± 0.24	3.4 ± 0.45
Iso-Butyric Acid	8.1	rancid butter cheese	0.74 ± 0.16	0.81 ± 0.079	0.7 ± 0.09	0.63 ± ±	1.02 ± 0.12
Butyric Acid	2.2	rancid cheese, sweet	2.27 ± 0.66	4 ± 0.23	2.37 ± 0.62	2.35 ± 0.42	3.96 ± 0.29
Ethyl Caprate	0.5	brandy fruity grape floral	0.24 ± 0.04	0.45 ± 0.01	0.21 ± 0.04	0.26 ± 0.04	0.45 ± 0.03
Iso-Valeric Acid	0.7	rancid cheese putrid	0.74 ± 0.11	0.95 ± 0.11	0.71 ± 0.15	0.67 ± 0.05	1.01 ± 0.11
Diethyl Succinate	1.2	Fruity, melon	0.09 ± 0.02	0.17 ± 0.02	0.05 ± 0.03	0.04 ± 0.01	0.16 ± 0.03
Valeric Acid		SWEATY	0.26 ± 0.03	0.49 ± 0.11	0.26 ± 0.03	0.26 ± 0.04	0.41 ± 0.04
2-Phenylethyl Acetate	1.8	rosy honey fruity	0.35 ± 0.06	0.55 ± 0.04	0.26 ± 0.04	0.27 ± 0.05	0.61 ± 0.05
2-Phenyl Ethanol	200	Rose, honey	11.88 ± 2.91	17.24 ± 0.69	16.68 ± 2.54	17.33 ± 2.28	17.98 ± 1.42
Octanoic Acid	10	Fatty, rancid, oily, soapy faint fruity	0.65 ± 0.08	1.07 ± 0.12	0.51 ± 0.14	0.65 ± 0.18	1.28 ± 0.09
Decanoic Acid	6	Fatty, rancid	0.84 ± 0.29	1.47 ± 0.07	0.64 ± 0.11	0.89 ± 0.19	1.52 ± 0.19

* Values are expressed as mg.L^-1 ^and are the average of 4 biological repeats ± standard deviation. Metabolites present at concentrations below the detection limit are indicated by "bd".

In general, the aroma compounds produced all showed a steady increase in concentration in the synthetic must over time, although the most active period of aroma compound accumulation appears to be in the earlier stages of fermentation. For the most part, compounds such as methanol, isoamyl alcohol, butanol, ethyl caprylate are only detectable in the fermentation media by day 5 of fermentation (table 3), whereas others such as diethyl succinate can only be detected at the end of fermentation (table 4). In general, the higher alcohols and their corresponding esters are present throughout fermentation at the highest concentration in the medium (tables 2, 3, 4). The aroma profiles of the DV10 and EC1118 strain are very similar, while the BM45 and 285 strains also produce similar exometabolomic signatures. The aroma compounds that are proportionally the most variable between strains are propanol, isobutanol, ethyl caprylate, acetic acid, propionic acid, butyric acid, ethyl caprate, diethyl succinate, valeric acid, 2-phenylethyl acetate, octanoic- and decanoic acid, as well as ethyl lactate, which is completely absent in the BM45 and 285 strains (table 4).

### Microarray analysis

The divergent aroma profiles of the different strains were mirrored by variable gene expression patterns. Since the Affymetrix DNA chips used for the analysis were designed based on the sequence of the laboratory yeast BY4742, a primary concern related to the quality of the microarray data. Both the internal controls and the expression of housekeeping genes were in keeping with international MIAME compliancy standards. Most notably, variation between independent biological repeats was negligible, giving us confidence in the reliability and reproducibility of our microarray analysis. Furthermore, changes in gene expression during the course of fermentation matched up well to data from related microarray analysis for the EC1118 [17] and VIN13 strains [18].

Between different time points approximately 1000–1500 genes significantly increased or decreased in expression (within the criteria specified in the materials and methods section) for the five yeast strains in our study. At the time points considered, the variation in gene expression between the different strains was in the range of about 50–400 transcripts. Strains that appear to be most similar to one another on a gene expression level were the EC1118 and DV10 strains, as well as the BM45 and 285 strains. The VIN13 strain was least similar to any of the other four strains. This pattern is in line with the differences observed in aroma production for all of these strains.

Numerous and substantial changes in the expression of genes involved in pathways that lead to the production of volatile aroma compounds were evident both between strains at comparable stages of fermentation and for individual strains at different fermentative stages. To identify relevant transcriptional variation in the context of aroma compound production, PCA analysis and PLS1 and PLS2 models were constructed for the compounds in tables 2, 3, 4 using the transcriptomic data as X variables. Transcriptomic data from days 2 and 5 were used for modeling purposes as these time points represent the period when the accumulation rate of most aroma compounds is at a maximum. From these models, transcripts with a strong positive or negative loading were selected for further in depth statistical analysis. The corresponding ORFs, together with a brief annotation, are listed in the additional data files [see Additional data file 1].

The general intrastrain trend revealed a decrease in the transcript levels of enzymes involved in the synthesis of aromatic and branched-chain amino acids, while transcript levels encoding aldehyde and alcohol dehydrogenases, as well as certain acetyltransferases were generally increased. Fold changes for differentially expressed transcripts, both between different strains at either day 2 or day 5 of fermentation and between day 2 and day 5 in individual strains, can be viewed as additional material [see Additional data file 2].

### Multivariate analysis of metabolite concentrations and gene expression data

**Figure 4:**
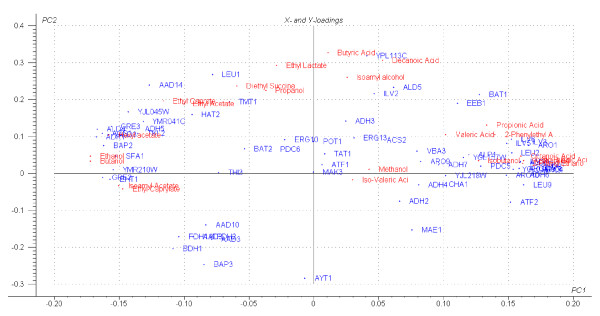
PLS2 scores and loadings plot of all X and Y variables considered in this study, plotted as coordinates on a PC1 and PC2 plane.

Figure 4 shows a PLS2 plot which depicts the variation/relationships between all the measured aroma compounds as well as the 70 genes selected for multivariate modeling purposes. These genes were selected due to their varying expression levels between different strains as well as different time points during fermentation. Also, we selected genes whose annotation suggested that they may have a role in aroma compound production, such as enzymes whose sequence suggests a role in redox reactions, central carbon metabolism, and amino acid uptake and metabolism (GO and MIPS classification).

**Figure 5:**
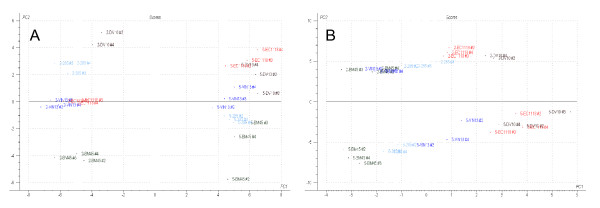
Scores plot for the ethyl caprylate (frame A) and octanoic acid (frame B) PLS1 models.

The X-Y scores and loading plots (figure 4) are clearly useful in representing the overall 'structure' of the entire dataset, and are pointing out possible connections between specific compounds/groups of compounds and certain genes. Likewise, scores plots proved a neat way of validating the general design and data generated by our experimental setup/process (figure 5). The samples of independent biological repeats for each of the 5 strains group together closely at both time points. All five strains also clearly segregate into two clusters based on the stage (time point) of fermentation. For example, in the first frame it is clear that the stage of fermentation is the major source of variation (PC1) and strain identity is the source of the second-greatest explained variation (PC2), while this pattern is reversed in frame B.

Of the 22 volatile aroma compound measured in this study, 13 were amenable to PLS1 modeling (using transcriptome data) based on our selected criteria for model validation (slope > 0.8; Y-var explained > 75%). The details of these models are summarized in a table that can be viewed as additional material [see Additional data file 3].

### Overexpression of selected genes

Of the genes listed in the tables presented in the supplementary material, five were chosen for in-depth analysis due to their significant contributions to the respective prediction models for several of the important higher alcohols and esters, as well as their amenability to easy cloning and vector construction. These genes were *BAT1, AAD10, AAD14, ACS1 *and *YMR210W*. *AAD10 *and *AAD14 *encode aryl alcohol dehydrogenases which are believed to be responsible for the putative role of degrading the complex aromatic compounds in grape must into their corresponding higher alcohols [7]. *BAT1 *encodes a mitochondrial branched-chain amino acid aminotransferase that is involved in catalyzing the first transamination step of the catabolic formation of fusel alcohols via the Ehrlich pathway [19]. The *YMR210 *gene codes for a putative acyltransferase enzyme (similar to *EEB1 *and *EHT1*) and is believed to play a role in medium-chain fatty acid ethyl ester biosynthesis. Lastly, the *ACS1 *gene (encoding an acetyl-coA synthetase isoform) codes for the enzyme responsible for the conversion of acetate to acetyl-coA, which is an intermediate or reactant in several of the aroma compound producing pathways [20].

An in-house *BAT1 *overexpressing strain was already available for use [21]. For the other 4 genes, a multi-copy overexpression plasmid-based cloning strategy was employed to allow for maximum gene expression and rapid characterization of the transformed VIN13 strains.

**Figure 6:**
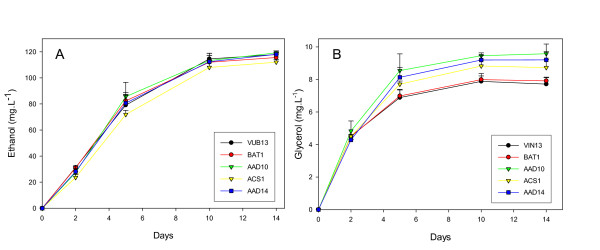
**Concentrations of ethanol (frame A) and glycerol (frame B) in the must during fermentation. **Values are the average of 4 independant repeats + standard deviation.

Fermentations were carried out as before with the 5 transformed cell lines and a VIN13 control. Samples for HPLC and GC-FID analysis were taken at the same time points, namely days 2, 5 and 14 of fermentation. No significant differences were observed regarding the glucose and fructose utilization of the overexpression strains during fermentation (Data not shown). Slight differences were found for ethanol production, while some changes in glycerol production were evident for the different strains (Figure 6).

**Figure 7:**
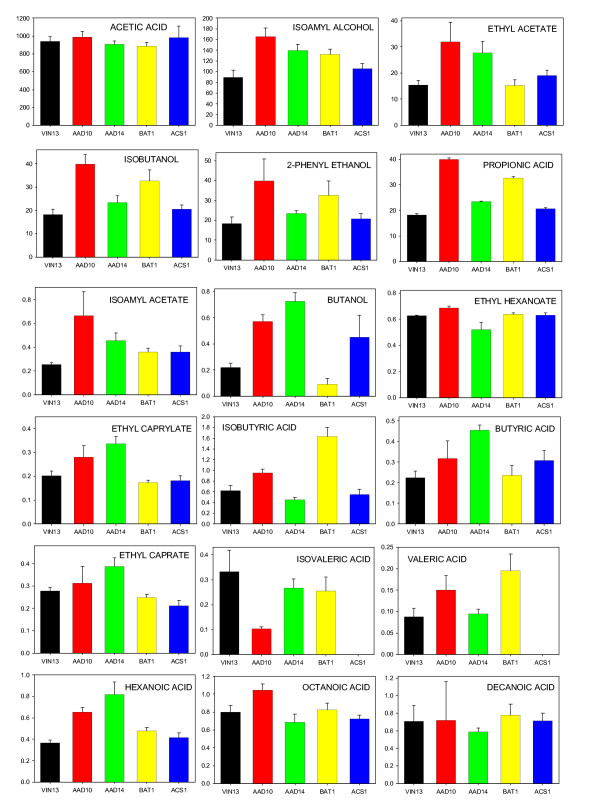
**Aroma compound production (μg.L^-1^) in MS300 fermentations carried out by VIN13 transformed with overexpression constructs. **Values are the average of 4 biological repeats + standard deviation.

Figure 7 depicts the aroma compound concentrations at the end of fermentation (day 14) only, as this is the most important time point from an enological perspective. Aroma profiles for days 2 and 5 can be viewed as additional data [see Additional data file 4].

Four of the five overexpressing strains showed significant changes in the aroma profiles produced at the end of fermentation. Only the *YMR210W *overexpressing strain did not show any changes, and is therefore not included in the figures below. We did not further investigate whether this absence of changes in aroma production is due to problems with the expression construct or reflects the absence of aroma–related activity of the gene product.

**Figure 8:**
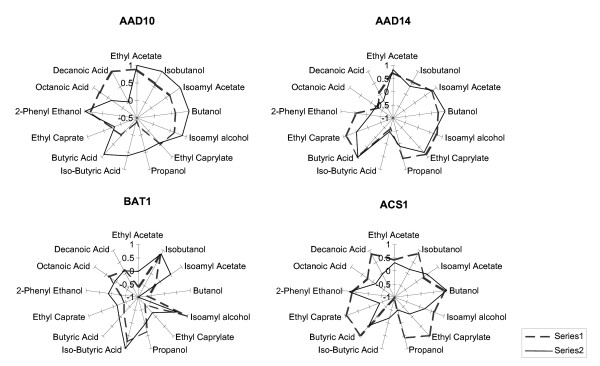
**Qualitative representation of relative real vs. predicted aroma compound levels in the four transformed VIN13 lines.** Dashed gray lines indicate predicted values and solid black lines indicate log-normalised values of real compound concentrations.

Significant differences were evident in the aroma profiles of the four transformed yeast strains under consideration. We investigated whether the observed changes in aroma compound concentrations at the end of fermentation can be reconciled with the anticipated changes based on multivariate prediction models. Figure 8 represents the qualitative alignment of real vs. predicted changes in aroma compound concentrations. Only aroma compounds with statistically reliable PLS models (test-set validation; slope >0.88; % RMSEP < 20) were taken into consideration. The dashed lines indicate the relative loading weights of each of the four genes (for each of the aroma compound models represented by the plot axes). The solid lines in the figures represent the log ratios of the actual aroma compound concentrations normalized to the VIN13 concentrations of the particular compound.

To clarify, the predicted influence of a given gene on a particular compound is represented on a scale from -1 to +1, based on statistical projections related to PLS loading weights. On this scale a value of -1 suggests a strong probability of significant concentration decreases of a given compound (for overexpression of the gene), while a value of +1 is indicative of a strong positive correlation between the expression levels of the gene of interest and the compound in question. A value of zero indicates no expected influence of gene expression on the relevant aroma compound.

Likewise, log-normalization was carried out on the actual metabolite concentrations measured in the overexpression strains to represent these values on a scale from -1 to 1, relative to the corresponding concentrations of the control fermentations. Figure 8 clearly shows that predicted and real changes overlapped significantly.

## Discussion

The aim of this study was to determine whether the transcription profiles of the various strains during fermentation could be reconciled with the volatile aroma compound production of these strains, and whether this comparative analysis could be used to predict the impact of individual gene expression levels on aroma compounds and profiles.

The data generated by the overexpression of four of the genes whose expression was statistically most significantly linked to the production of aroma profiles suggest that this approach has been successful. Indeed, overexpression of the selected genes had a far reaching impact on the aroma profiles produced by the fermenting yeast, and this impact was generally well aligned with the impact predicted from the comparative omics analysis. Indeed, the data aligned better than we, considering the significant challenges when approaching complex systems, had expected. Our data show that the metabolic changes observed upon overexpression of three of the four genes, *AAD10*, *AAD14 *and *BAT1*, were very significantly aligned with the changes that were predicted from the alignment of transcriptome and metabolome data alone. The predictions, as can be seen from the alignment of predicted vs. observed changes in metabolite levels in a qualitative manner, indeed proved fairly reliable. The model was able to assign positive and negative influences on a particular compound with relative accuracy. Although the extent/magnitude of the increase/decrease is not always well aligned with model values, the absolute direction of the change holds true in most cases. An absolute alignment would not be expected, since the level of expression in a plasmid-based system can not be adjusted to the differences of expression observed between the different strains. In the case of *AAD10*, only the influence of the overexpression on decanoic acid was not in line with the projection. Predictions for *AAD14 *and *BAT1 *were well matched with the observed changes in metabolite profiles. Predicted and real changes did not match satisfactorily in only one case, *ACS1*. Nevertheless, even in this case, eight out of the thirteen compounds evolved in the predicted direction. It should also be noted that the expression of this gene had generally a less severe impact on changes in the aroma profile than those of the other three genes.

Considering the complexity of the system, the rate of success achieved in this study can be considered as highly significant. To our knowledge, this is the first report to exploit such an intra- and interstrain comparative approach to identify genes that play a significant role in a complex metabolic network.

While we were clearly able to identify genes with significant impact on aroma compound production in a specific industrial environment, and which in some cases had not been previously directly linked to these pathways, the data do not allow a firm conclusion on the exact metabolic role of these genes. Indeed, the vast number of significant changes to metabolite levels makes it difficult to identify the specific 'point of influence' of any overexpressed gene in a given pathway.

The increases/decreases in specific volatile compounds seen for the VIN13(pBAT1-s) strain is in keeping with the results reported in colombar fermentations [21]. The two AAD gene overexpressing strains also showed interesting trends: Both strains produced higher levels (at comparable concentrations) of isoamyl alcohol, ethyl acetate, butanol, ethyl caprylate, ethyl caprate and hexanoic acid. However, noticeable differences can be seen in the levels of isobutanol, 2-phenyl ethanol, propionic acid, isoamyl acetate, ethyl hexanoate, isobutyric acid and isovaleric acid, relative to the control and to one another. This is indicative of the potential for the AAD genes to have overlapping yet distinct functional roles in the pathways leading to higher alcohol and ester production.

Overexpression of the *ACS1 *gene did not lead to such numerous and substantial increases/decreases in volatile production as was the case for the other three genes. Interestingly, valeric and isovaleric acid were below detection levels in these fermentations. Concentrations of isoamyl acetate, ethyl acetate, butanol and butyric acid were significantly higher, and ethyl caprate lower relative to control fermentations.

On the whole though, our analysis shows that the cross-comparison of gene expression data with metabolite levels has the potential to identify points of interest on a genomic scale. This also opens new possibilities to design improved yeast enhancement strategies for optimized aroma production and fermentation performance.

### Other genes of interest

Many other genes showed significant variation in expression between different strains and/or time points, as well as high loadings on PLS models and strong negative or positive correlations with specific aroma compounds. These genes encode enzymes that either are known to participate in aroma compound production, or have activities (either experimentally proven or suggested through sequence alignments) that could suggest such roles. Here we discuss some of the most relevant of these enzymes, which fall into several categories, either according to their place in a specific metabolic pathway such as the metabolisms of branched chain amino acids or of aromatic amino acids, or based on their specific activity such as dehydrogenases (in particular aldehyde and alcohol dehydrogenases) and acetyl transferases.

Of the enzymes involved in branched chain amino acid metabolism, *BAT1 *has been discussed above. Other genes that encode enzymes in this pathway and that were identified in our study for their strong statistical link between expression levels and the production of specific compounds include *LEU2*, encoding a beta-isopropylmalate dehydrogenase that catalyzes the third step in the leucine biosynthesis pathway, and, to a lesser degree, *LEU1*, which encodes an isopropylmalate isomerase [22,23]. Both of these genes showed a significant statistical correlation with compounds such as isobutanol. Of the genes involved in the metabolism of isoleucine and valine (Ilv), only *ILV5*, which encodes an acetohydroxyacid reductoisomerase involved in branched-chain amino acid biosynthesis [24], showed a very strong positive correlation with almost all of the compounds analysed here, and, interestingly, a negative correlation with ethanol, suggesting that this gene could be an interesting target for metabolic engineering.

While *BAT1 *expression showed a significant positive correlation with a large number of the volatile compounds measured in our study, the cytosolic isoform (*BAT2*) of this enzyme showed no significant correlations with any of these aroma compounds. Although this isoform is supposedly highly expressed during stationary phase and repressed during the logarithmic phase, *BAT2 *expression levels in our study were found to stay constant, if not to decrease slightly upon entry into stationary phase in comparison to the exponential phase at day 2. In addition, *BAT2 *expression levels were generally considerably lower throughout fermentation when compared to *BAT1*.

Of the genes involved in aromatic amino acid metabolism, three, *ARO1*, which encodes a pentafunctional arom protein, *ARO7*, which encodes a chorismate mutase responsible for the conversion of chorismate to prephenate and *ARO8*, which codes for an aromatic aminotransferase showed statistically significant correlations between expression levels and metabolite production [25,26]. All three genes showed a modest positive correlation (r^2 ^= 0.7) with 2-phenyl ethanol and mild negative correlations with all the other compounds. Only octanoic acid showed a very strong (r^2 ^= 0.82) negative correlation with *ARO8 *expression at day 2 of fermentation. Despite its seemingly crucial role, *ARO10*, which encodes a phenylpyruvate decarboxylase corresponding to the first specific step in the Ehrlich pathway did not show any noteworthy correlations between its expression and any of the volatile compounds in our study [27]. Of course the possibility of translational or post-translational control of activity cannot be excluded.

Several specific enzyme activities were also overrepresented in our list. Such enzymes include many dehydrogensases. Aldehyde and alcohol dehydrogenases such as those encoded by *ALD5*, *ALD6*, *ADH6 *and *ADH7 *showed a substantial decline in expression levels between days 2 and 5 of fermentation, while others (such as *ALD3*, *ALD4*, *ADH2 *and *ADH5*) increased during this time. The distinct expression patterns during fermentation reflects the different regulatory mechanisms governing the expression of these genes (i.e. expression of *ALD3 *is glucose-repressed and stress-induced) and suggests that the different ALD gene products have specific roles during different stages of fermentation [28].

*ALD4 *and *ALD5 *(mitochondrial), and *ALD3 *and *ALD6 *(cytoplasmic) encode aldehyde dehydrogenases involved in the conversion of acetaldehyde to acetate [29].

*ALD4 *encodes a mitochondrial aldehyde dehydrogenase (utilizing NADP+ or NAD+) that is required for growth on ethanol and conversion of acetaldehyde to acetate [29]. Expression of *ALD4 *is also glucose repressed, and increases 2–4 -fold from day 2 to 5 of fermentation. *ALD4 *expression shows a very strong correlation to the amount of hexyl acetate (R^2 ^= 0.82) produced by the fermenting yeast, as well as to ethyl acetate (0.77), isoamyl alcohol (0.91) and isoamyl acetate (0.85).

*ALD6 *encodes a constitutively expressed cytosolic aldehyde dehydrogenase (utilizes NADP+ as the preferred coenzyme) and is required for conversion of acetaldehyde to acetate [30]. Not surprisingly, *ALD6 *expression showed a very strong positive correlation to the levels of acetic acid produced by the fermenting cells (0.92). Also, expression was very strongly inversely correlated to ethanol production (R^2 ^= 0.81). Interestingly, fairly strong positive correlations were also evident for 2-phenyl ethanol (R^2 ^= 0.79) and 2-phenyl ethyl acetate (R^2 ^= 0.67).

*ADH6 *encodes an NADPH-dependent cinnamyl alcohol dehydrogenase family member with broad substrate specificity [31]. Expression was correlated very strongly with isobutanol levels (0.81), isobutyric acid (0.86), propionic acid (0.81), acetic acid (0.87) and 2-phenyl ethanol (0.92). *ADH4*, *ADH5 *and *ADH7 *on the other hand showed only modest correlations with the above-mentioned, or any other aroma compounds for that matter.

With respect to the aryl alcohol dehydrogenase family of genes, the transcripts for *AAD3*, *AAD10 *and *AAD14 *showed the greatest variation in expression, both on an intra- and interstrain level. Expression of *AAD10 *and *AAD14*, for example, was increased more than twofold in most of the strains at day 5 relative to day 2 of fermentation. No distinct physiological role has been established for the products of these genes [7], but it is reasonable to suspect that the consistent increase in their respective transcript levels during the course of fermentation could be associated with the increase in one or several of the long chain alcohols or their acid counterparts as fermentation progresses (tables 2, 3).

This hypothesis is supported by the data generated through the overexpression of these genes. Indeed, overexpression yielded changes to the aroma profile that were very similar to those predicted from the alignment of transcriptome and metabolome data sets. The expression of *AAD10 *showed weak yet significant positive correlations with a number of the aroma compounds. Expression of *AAD14 *between different strains and time points was also highly variable. Highest expression levels were noted for the DV10 strain, and significant positive correlations with ethyl acetate (0.67) and ethyl caprate (0.74) were observed for this gene.

Acetyl transferases are another family of enzymes of relevance to aroma compound metabolism [32]. However, neither *ATF1 *nor *ATF2*, the two most prominent alcohol acetyl transferases, showed statistically strong correlations between expression levels and metabolite production. *EEB1*, on the other hand, which encodes an acyl-coenzymeA:ethanol O-acyltransferase and is responsible for the major part of medium-chain fatty acid ethyl ester biosynthesis during fermentation [33], showed weak negative correlations with ethanol and other higher alcohols, and a strong positive correlation for 2-phenylethyl acetate (0.9) as well as octanoic acid (0.78). It is tempting to speculate that Eeb1p may thus be largely responsible for the acetylation of 2-phenyl ethanol to produce 2-phenylethyl acetate.

*EHT1 *encodes an acyl-coenzymeA:ethanol O-acyltransferase that plays a role in medium-chain fatty acid ethyl ester biosynthesis, but also contains a known esterase activity [33]. *EHT1 *expression increased somewhat as fermentation progressed and inter-strain expression at both day 2 and 5 of fermentation varied significantly. Interestingly, *EHT1 *expression showed a fairly strong inverse correlated with 2-phenylethyl acetate (R^2 ^= 0.74) and octanoic acid (R^2 ^= 0.75), as well as a weaker yet significant inverse correlation with decanoic acid (R^2 ^= 0.59). This could indicate that the esterase activity of Eht1p could predominate under certain conditions.

*YMR210W *encodes a putative acyltransferase with similarity to both Eeb1p and Eht1p, and may have a minor role in medium-chain fatty acid ethyl ester biosynthesis [33]. Expression was positively correlated with ethyl acetate (0.74), ethyl caprylate (0.85) and isoamyl acetate (0.78).

In addition to these relatively well studied acetyltransferases, the mRNA levels of the *AYT1 *gene, encoding a transferase of unknown substrate specificity, also showed considerable variation at different fermentative stages [34].

## Conclusion

The impact of these individual genes on aroma compound metabolism has to be assessed individually. However, from the data presented here, it is clear that an analysis based on the comparison of transcriptome and metabolome data derived from different commercial yeast strains can help to identify genes that most significantly impact a metabolic network in specific environmental and industrial conditions. Our over-expression analysis of five genes that were randomly selected from the list of ORFs identified for their statistically significant impact on aroma production also clearly suggests that the method has significant predictive power regarding the reorientation of metabolic flux through the network in response to changes in gene expression levels. Indeed, for four out of five selected genes, *BAT1*, *AAD10*, *AAD14 *and *ACS1*, the match between predicted and real changes is highly significant. This is the first study linking metabolic networks to transcriptome analysis through the comparative analysis of different wine yeast strains.

## Methods

### Strains. media and culture conditions

The yeast strains used in this study are listed in table 5. All are diploid *Saccharomyces cerevisiae *strains used in industrial wine fermentations. Yeast cells were cultivated at 30°C in YPD synthetic media 1% yeast extract (Biolab, South Africa), 2% peptone (Fluka, Germany), 2% glucose (Sigma, Germany). Solid medium was supplemented with 2% agar (Biolab, South Africa).

**Table 5:** Strains used in this study

Strain	Source/Reference
VIN13	Anchor Yeast, South Africa
EC1118	Lallemand Inc., Montréal, Canada
BM45	Lallemand Inc., Montréal, Canada
285	Lallemand Inc., Montréal, Canada
DV10	Lallemand Inc., Montréal, Canada
VIN13(pBAT1-s)	Lilly *et al*., 2006

### Fermentation media

Fermentation experiments were carried out with synthetic must MS300 which approximates to a natural grape must as previously described [35]. The medium contained 125 g/L glucose and 125 g/L fructose, and the pH was buffered at 3.3 with NaOH.

### Fermentation conditions

All fermentations were carried out under microaerophilic conditions in 100 ml glass bottles (containing 80 ml of the medium) sealed with rubber stoppers with a CO_2 _outlet. The fermentation temperature was approximately 22°C and no continuous stirring was performed during the course of the fermentation. Fermentation bottles were inoculated with YPD cultures in the logarithmic growth phase (around OD_600 _= 1) to an OD_600 _of 0.1 (i.e. a final cell density of approximately 10^6 ^cfu.ml^-1^). The cells from the YPD pre-cultures were briefly centrifuged and resuspended in MS300 to avoid carryover of YPD to the fermentation media. The fermentations followed a time course of 14 days and the bottles were weighed daily to assess CO_2 _release and the progress of fermentation. Samples of the fermentation media and cells were taken at days 2, 5 and 14 as representative of the exponential, early stationary and late stationary growth phases respectively. It should be stressed that early stationary phase in these conditions is metabolically active, since growth arrest is due to ethanol toxicity. Sugar levels and fermentative activity are still high at this stage.

### Growth measurement

Cell proliferation (i.e. growth) was determined spectrophotometrically (Powerwave_X_, Bio-Tek Instruments) by measuring the optical density (at 600 nm) of 200 μl samples of the suspensions over the 14 day experimental period.

### Analytical methods – HPLC

Culture supernatants were obtained from the cell-free upper layers of the fermentation media. For the purposes of glucose determination and carbon recovery, culture supernatants and starting media were analyzed by high performance liquid chromatography (HPLC) on an AMINEX HPX-87H ion exchange column using 5 mM H_2_SO_4 _as the mobile phase. Agilent RID and UV detectors were used in tandem for peak detection and quantification. Analysis was carried out using the HPChemstation software package.

### Analytical methods – GC-FID

Each 5 ml sample of synthetic must taken during fermentation was spiked with an internal standard of 4-methyl-2-pentanol to a final concentration of 10 mg.l^-1^. To each of these samples 1 ml of solvent (diethyl ether) was added and the tubes sonicated for 5 minutes. The top layer in each tube was separated by centrifugation at 3000 rpm for 5 minutes and the extract analyzed. After mixing, 3 μl of each sample was injected into the gas chromatograph (GC). All extractions were done in triplicate.

The analysis of volatile compounds was carried out on a Hewlett Packard 5890 Series II GC coupled to an HP 7673 auto-sampler and injector and an HP 3396A integrator. The column used was a Lab Alliance organic-coated, fused silica capillary with dimensions of 60 m × 0.32 mm internal diameter with a 0.5 μm coating thickness. The injector temperature was set to 200°C, the split ratio to 20:1 and the flow rate to 15 ml.min^-1^, with hydrogen used as the carrier gas for a flame ionisation detector held at 250°C. The oven temperature was increased from 35°C to 230°C at a ramp of 3°C min^-1^.

Internal standards (Merck, Cape Town) were used to calibrate the machine for each of the compounds measured.

### Statistical analysis of metabolite data

T-tests and anova analyses were conducted using Statistica (version 7). HCL and KMC clustering were carried out using TIGR MeV v2.2 [36].

### Microarray analysis

Sampling of cells from fermentations and total RNA extraction was performed as described [37]. Probe preparation and hybridization to Affymetrix Genechip^® ^microarrays were performed according to Affymetrix instructions, starting with 6 μg of total RNA. Results for each strain and time point were derived from three independent culture replicates. The quality of total RNA, cDNA, cRNA and fragmented cRNA were confirmed using the Agilent Bioanalyzer 2100.

### Transcriptomics data acquisition and statistical analysis

Acquisition and quantification of array images and data filtering were performed using Affymetrix GeneChip^® ^Operating Software (GCOS) version 1.4. All arrays were scaled to a target value of 500 using the average signal from all gene features using GCOS. Genes with expression values below 12 were set to 12 + the expression value as previously described in order to eliminate insignificant variations [38].

Variable (gene) selection is important for the successful analysis of gene expression data since most of the genes are unchanged and irrelevant to the prediction and analysis of phenotypic measurements. These non-informative genes should be removed before further analysis. One approach is by significance analysis of microarrays [39]. Determination of differential gene expression between experimental parameters was conducted using SAM (Significance Analysis of Microarrays) version 2. The two-class, unpaired setting was used and genes with a Q value less than 0.5 were considered differentially expressed. Only genes with a fold change greater than 2 (positive or negative) for inter- or intra- strain comparisons were taken into consideration.

### Multivariate data analysis

In terms of design, the samples represent the different fermentations (three independent replicates for each of the five strains) at different time points. The variables considered are the expression levels of the pre-selected genes (genes with a potential and established role in aroma compound metabolism according to GO and MIPS functional classification) as well as aroma compound concentrations in the synthetic must. The patterns within the different sets of data were investigated by principal-component analysis (PCA), while the correlations between different sets of data were determined by using partial least-squares (PLS) regression (The Unscrambler; Camo Inc., Corvallis, Oreg.). PCA is a bilinear modeling method which gives a visually interpretable overview of the main information in large, multidimensional datasets. By plotting the principal components it is possible to view statistical relationships between different variables in complex datasets and detect and interpret sample groupings, similarities or differences, as well as the relationships between the different variables [40].

PLS regression is a bilinear modeling method for identifying the variations in a data matrix for explanatory or predictive purposes [41]. By plotting the first PLS components one can view main associations between X variables and Y variables and also relationships within X data and within Y data. PLS2 analysis was conducted using all X and Y variables considered in our study. For predictive purposes, PLS1 models were constructed for individual Y variables to increase model-specificity and reliability.

The data were analyzed by using test-set validation with centered data and the variables were weighted according to their standard deviations. One strain was used as the test segment at each of the time points. Day 2 and 5 data were considered together as representative of the full scope of fermentation variability as the period from the start of fermentation until day 5 represents the period of maximum aroma compound production.

The Y variables were the respective aroma compounds measured and the X variables were the gene expression levels of the gene set that was pre-selected for analysis [42]. Genes were selected based on known or putative functions related to amino acid transport, metabolism, regulation etc, as well as other enzymatic or regulatory activity in pathways leading to the production of higher alcohols and esters. The same set of genes (X variables) was used for each of the different PLS1 models.

### Overexpression constructs and transformation of VIN13

All plasmids used in this study are listed in table 6. Standard procedures for the isolation, cloning and modification of DNA were used throughout this study [43,44]. All enzymes for cloning, restriction digest and ligation reactions were obtained from Roche Diagnostics (Randburg, South Africa) and used according to supplier specifications.

**Table 6:** Description of plasmids used in this study

Plasmid Name	Relevant genotype	Reference
pDM-PhR-YMR210W	2 μ LEU2 TEF1_P _PhR322 TEF1_T _PGK_P _YMR210W PGK_T_	This study
pDM-PhR-AAD10	2 μ LEU2 TEF1_P _PhR322 TEF1_T _PGK_P _AAD10 PGK_T_	This study
pDM-PhR-AAD14	2 μ LEU2 TEF1_P _PhR322 TEF1_T _PGK_P _AAD14 PGK_T_	This study
pDM-PhR-ACS1	2 μ LEU2 TEF1_P _PhR322 TEF1_T _PGK_P _ACS1 PGK_T_	This study

The primers listed in table 7 were used to amplify the coding regions of the various genes by the PCR technique. Genomic DNA from the DV10 strain was used as the template. *Eshericia coli *DH5α (GIBCO-BRL/Life Technologies) was used as the host for the construction and propagation of the plasmids listed in table 6. Sequencing of all plasmids was carried out on an ABI PRISM automated sequencer. All plasmids contain the dominant marker PhR conferring phleomicin resistance (PhR), and were transformed into host VIN13 cells via electroporation [21,45].

**Table 7:** Sequences of the primers synthesized in order to amplify genes relevant to the present study

Primer Name	Sequence (5'-3')
PhR322F	GATCCACGTCGGTACCCGGGGGATC
PhR322R	GATCGCGATCGCAAGCTTGCAAATTAAAGCC
YMR210f	AACGCTGGTAAACTTCCAGAGA
YMR210r	GGCGAAGCTTTTCACGTTTT
AAD10f	ATGCTTTTTACCAAGCAGGC
AAD10r	CATCAAACTGTGTGTGTAAGCG
AAD14f	ACCAATTAGCTGAACGGCTTTG
AAD14r	ATTTGCACACACTCGGTGGATA
ACS1f	AAAGACATTGCCCACTGTGCT
ACS1r	CACGAAAAAAAAAAAGTCGTCA

## Authors' contributions

DR carried out the experimental work and contributed to the experimental design and data analysis. She also drafted the manuscript. TN contributed to the statistical analysis of the data. FFB conceived of the study and participated in its design and coordination. All authors read and approved of the final manuscript.

## Supplementary Material

Additional file 1 **Identified ORFs and functional description.** The table lists all ORFs whose expression pattern correlated significantly with aroma compound production. A short functional annotation is also provided. Click here for file

Additional file 2 **Inter- and intra-strain differences in expression levels of all identified ORFs.** The three tables show the differences in expression levels of identified ORFs between different strains at day 2 (Table 1) and at day 5 (Table 2) of fermentation, and differences between day 2 and day 5 in individual strains (Table 3). Click here for file

Additional file 3 **Summaries of PLS1 models.** The table summarises PLS1 models used for interpretation and selection of genes for overexpression. Click here for file

Additional file 4 **Comparison between metabolites produced in the strains overexpressing individual ORFs.** The table shows a comparison between the volatile aroma metabolites that are produced in the strains overexpressing individual ORFs at day 2 and day 5 of fermentation. Click here for file
