# Supplementary material for: Linking gene regulation and the exo-metabolome: A comparative transcriptomics approach to identify genes that impact on the production of volatile aroma compounds in yeast
Source: BMC Genomics. 2008 Nov 7;9:530. doi: 10.1186/1471-2164-9-530 (PMC2585593; doi:10.1186/1471-2164-9-530)
Supplement: Additional file 1 — Identified ORFs and functional description. The table lists all ORFs whose expression pattern correlated significantly with aroma compound production. A short functional annotation is also provided. [file 1471-2164-9-530-S1.doc]

**Additional data file 1:**

**Identified ORFs and functional description**

| GENE NAME | SYSTEMATIC NAME | FUNCTIONAL DESCRIPTION (BRIEF) |
| --- | --- | --- |
| AAD3 | YCR107W | Putative aryl-alcohol dehydrogenase with similarity to P. chrysosporium aryl-alcohol dehydrogenase; mutational analysis has not yet revealed a physiological role |
| POT1 | YIL160C | 3-ketoacyl-CoA thiolase with broad chain length specificity, cleaves 3-ketoacyl-CoA into acyl-CoA and acetyl-CoA during beta-oxidation of fatty acids |
| LEU2 | YCL018W | Beta-isopropylmalate dehydrogenase, catalyzes the third step in the leucine biosynthesis pathway |
| ALD3 | YMR169C | Cytoplasmic aldehyde dehydrogenase, involved in beta-alanine synthesis; uses NAD+ as the preferred coenzyme; very similar to Ald2p; expression is induced by stress and repressed by glucose |
| SFA1 | YDL168W | Bifunctional enzyme containing both alcohol dehydrogenase and glutathione-dependent formaldehyde dehydrogenase activities, functions in formaldehyde detoxification and formation of long chain and complex alcohols |
| EEB1 | YPL095C | Acyl-coenzymeA:ethanol O-acyltransferase responsible for the major part of medium-chain fatty acid ethyl ester biosynthesis during fermentation; possesses short chain esterase activity |
| YJL218W | YJL218W | Putative protein of unknown function, similar to bacterial galactoside O-acetyltransferases; induced by oleate in an OAF1/PIP2-dependent manner |
| ARO1 | YDR127W | Pentafunctional arom protein, catalyzes steps 2 through 6 in the biosynthesis of chorismate, which is a precursor to aromatic amino acids |
| ADH6 | YMR318C | NADPH-dependent cinnamyl alcohol dehydrogenase family member with broad substrate specificity; may be involved in fusel alcohol synthesis or in aldehyde tolerance |
| ATF2 | YGR177C | Alcohol acetyltransferase, may play a role in steroid detoxification; forms volatile esters during fermentation, which is important in brewing |
| ARO10 | YDR380W | Phenylpyruvate decarboxylase, catalyzes decarboxylation of phenylpyruvate to phenylacetaldehyde, which is the first specific step in the Ehrlich pathway |
| PDC6 | YGR087C | Minor isoform of pyruvate decarboxylase, key enzyme in alcoholic fermentation, decarboxylates pyruvate to acetaldehyde, regulation is glucose- and ethanol-dependent, involved in amino acid catabolism |
| ALP1 | YNL270C | Basic amino acid transporter, involved in uptake of cationic amino acids |
| ALD5 | YER073W | Mitochondrial aldehyde dehydrogenase, involved in regulation or biosynthesis of electron transport chain components and acetate formation; activated by K+; utilizes NADP+ as the preferred coenzyme; constitutively expressed |
| ARO7 | YPR060C | Chorismate mutase, catalyzes the conversion of chorismate to prephenate to initiate the tyrosine/phenylalanine-specific branch of aromatic amino acid biosynthesis |
| ADH3 | YMR083W | Mitochondrial alcohol dehydrogenase isozyme III; involved in the shuttling of mitochondrial NADH to the cytosol under anaerobic conditions and ethanol production |
| ACS1 | YAL054C | Acetyl-coA synthetase isoform which, along with Acs2p, is the nuclear source of acetyl-coA for histone acetlyation; expressed during growth on nonfermentable carbon sources and under aerobic conditions |
| GRE2 | YOL151W | NADPH-dependent methylglyoxal reductase (D-lactaldehyde dehydrogenase); stress induced (osmotic, ionic, oxidative, heat shock and heavy metals); regulated by the HOG pathway |
| HPA3 | YEL066W | D-Amino acid N-acetyltransferase; similar to Hpa2p, acetylates histones weakly in vitro |
| BAP3 | YDR046C | Amino acid permease involved in the uptake of cysteine, leucine, isoleucine and valine |
| HAT2 | YEL056W | Subunit of the Hat1p-Hat2p histone acetyltransferase complex; |
| ILV5 | YLR355C | Acetohydroxyacid reductoisomerase, mitochondrial protein involved in branched-chain amino acid biosynthesis, also required for maintenance of wild-type mitochondrial DNA |
| ARO4 | YBR249C | 3-deoxy-D-arabino-heptulosonate-7-phosphate (DAHP) synthase, catalyzes the first step in aromatic amino acid biosynthesis and is feedback-inhibited by tyrosine or high concentrations of phenylalanine or tryptophan |
| ILV3 | YJR016C | Dihydroxyacid dehydratase, catalyzes third step in the common pathway leading to biosynthesis of branched-chain amino acids |
| ADH2 | YMR303C | Glucose-repressible alcohol dehydrogenase II, catalyzes the conversion of ethanol to acetaldehyde; involved in the production of certain carboxylate esters; regulated by ADR1 |
| VBA3 | YCL069W | Permease of basic amino acids in the vacuolar membrane /// Hypothetical protein |
| FDH1 /// FDH2 | YOR388C | NAD(+)-dependent formate dehydrogenase, may protect cells from exogenous formate |
| AAD10 | YJR155W | Putative aryl-alcohol dehydrogenase with similarity to P. chrysosporium aryl-alcohol dehydrogenase; mutational analysis has not yet revealed a physiological role |
| YJL045W | YJL045W | Minor succinate dehydrogenase isozyme; homologous to Sdh1p, the major isozyme reponsible for the oxidation of succinate and transfer of electrons to ubiquinone; induced during the diauxic shift in a Cat8p-dependent manner |
| PDC5 | YLR134W | Minor isoform of pyruvate decarboxylase, key enzyme in alcoholic fermentation, decarboxylates pyruvate to acetaldehyde, regulation is glucose- and ethanol-dependent, repressed by thiamine, involved in amino acid catabolism |
| ACS2 | YLR153C | Acetyl-coA synthetase isoform which, along with Acs1p, is the nuclear source of acetyl-coA for histone acetlyation; required for growth on glucose; expressed under anaerobic conditions |
| BAP2 | YBR068C | High-affinity leucine permease, functions as a branched-chain amino acid permease involved in the uptake of leucine, isoleucine and valine |
| ERG10 | YPL028W | Acetyl-CoA C-acetyltransferase (acetoacetyl-CoA thiolase), cytosolic enzyme that transfers an acetyl group from one acetyl-CoA molecule to another, forming acetoacetyl-CoA; involved in the first step in mevalonate biosynthesis |
| ARO9 | YHR137W | Aromatic aminotransferase, catalyzes the first step of tryptophan, phenylalanine, and tyrosine catabolism |
| YMR041C | YMR041C | Putative protein of unknown function with similarity to aldo/keto reductases; YMR041C is not an essential gene |
| ARO8 | YGL202W | Aromatic aminotransferase, expression is regulated by general control of amino acid biosynthesis |
| ERG13 | YML126C | 3-hydroxy-3-methylglutaryl-CoA (HMG-CoA) synthase, catalyzes the formation of HMG-CoA from acetyl-CoA and acetoacetyl-CoA; involved in the second step in mevalonate biosynthesis |
| ADR1 | YDR216W | Carbon source-responsive zinc-finger transcription factor, required for transcription of the glucose-repressed gene ADH2, of peroxisomal protein genes, and of genes required for ethanol, glycerol, and fatty acid utilization |
| TAT1 | YBR069C | Amino acid transport protein for valine, leucine, isoleucine, and tyrosine, low-affinity tryptophan and histidine transporter |
| ILV1 | YER086W | Threonine deaminase, catalyzes the first step in isoleucine biosynthesis; expression is under general amino acid control |
| ALD4 | YOR374W | Mitochondrial aldehyde dehydrogenase, required for growth on ethanol and conversion of acetaldehyde to acetate; activity is K+ dependent; utilizes NADP+ or NAD+ equally as coenzymes; expression is glucose repressed |
| MAE1 | YKL029C | Mitochondrial malic enzyme, catalyzes the oxidative decarboxylation of malate to pyruvate, which is a key intermediate in sugar metabolism and a precursor for synthesis of several amino acids |
| BAT2 | YJR148W | Cytosolic branched-chain amino acid aminotransferase; highly expressed during stationary phase and repressed during logarithmic phase |
| BDH1 | YAL060W | NAD-dependent (2R,3R)-2,3-butanediol dehydrogenase, a zinc-containing medium-chain alcohol dehydrogenase, produces 2,3-butanediol from acetoin during fermentation |
| LEU1 | YGL009C | Isopropylmalate isomerase, catalyzes the second step in the leucine biosynthesis pathway |
| YMR210W | YMR210W | Putative acyltransferase with similarity to Eeb1p and Eht1p, has a minor role in medium-chain fatty acid ethyl ester biosynthesis; may be involved in lipid metabolism and detoxification |
| YGL039W | YGL039W | Oxidoreductase, catalyzes NADPH-dependent reduction of the bicyclic diketone bicyclo[2.2.2]octane-2,6-dione (BCO2,6D) to the chiral ketoalcohol (1R,4S,6S)-6-hydroxybicyclo[2.2.2]octane-2-one (BCO2one6ol) |
| YGL157W | YGL157W | Oxidoreductase, catalyzes NADPH-dependent reduction of the bicyclic diketone bicyclo[2.2.2]octane-2,6-dione (BCO2,6D) to the chiral ketoalcohol (1R,4S,6S)-6-hydroxybicyclo[2.2.2]octane-2-one (BCO2one6ol) |
| THI3 | YDL080C | Probable decarboxylase, required for expression of enzymes involved in thiamine biosynthesis; may have a role in catabolism of amino acids to long-chain and complex alcohols |
| ADH7 | YCR105W | NADPH-dependent cinnamyl alcohol dehydrogenase family member with broad substrate specificity; may be involved in fusel alcohol synthesis |
| AYT1 | YLL063C | Acetyltransferase; catalyzes trichothecene 3-O-acetylation, suggesting a possible role in trichothecene biosynthesis |
| TKL2 | YBR117C | Transketolase; catalyzes conversion of xylulose-5-phosphate and ribose-5-phosphate to sedoheptulose-7-phosphate and glyceraldehyde-3-phosphate in the pentose phosphate pathway; needed for synthesis of aromatic amino acids |
| TMT1 | YER175C | Trans-aconitate methyltransferase, cytosolic enzyme that catalyzes the methyl esterification of 3-isopropylmalate, an intermediate of the leucine biosynthetic pathway, and trans-aconitate, which inhibits the citric acid cycle |
| ADH4 | YGL256W | Alcohol dehydrogenase type IV, dimeric enzyme demonstrated to be zinc-dependent despite sequence similarity to iron-activated alcohol dehydrogenases |
| ALD6 | YPL061W | Cytosolic aldehyde dehydrogenase, activated by Mg2+ and utilizes NADP+ as the preferred coenzyme; required for conversion of acetaldehyde to acetate; constitutively expressed |
| CHA1 | YCL064C | Catabolic L-serine (L-threonine) deaminase, catalyzes the degradation of both L-serine and L-threonine; required to use serine or threonine as the sole nitrogen source, transcriptionally induced by serine and threonine |
| TKL1 | YPR074C | Transketolase; catalyzes conversion of xylulose-5-phosphate and ribose-5-phosphate to sedoheptulose-7-phosphate and glyceraldehyde-3-phosphate in the pentose phosphate pathway; needed for synthesis of aromatic amino acids |
| BAT1 | YHR208W | Mitochondrial branched-chain amino acid aminotransferase, homolog of murine ECA39; highly expressed during logarithmic phase and repressed during stationary phase |
| GRE3 | YHR104W | Aldose reductase involved in methylglyoxal, d-xylose and arabinose metabolism; stress induced (osmotic, ionic, oxidative, heat shock, starvation and heavy metals); regulated by the HOG pathway |
| EHT1 | YBR177C | Acyl-coenzymeA:ethanol O-acyltransferase that plays a minor role in medium-chain fatty acid ethyl ester biosynthesis; contains esterase activity; localizes to lipid particles and the mitochondrial outer membrane |
| ADH5 | YBR145W | Alcohol dehydrogenase isoenzyme V; involved in ethanol production |
| ILV6 | YCL009C | Regulatory subunit of acetolactate synthase, which catalyzes the first step of branched-chain amino acid biosynthesis; enhances activity of the Ilv2p catalytic subunit, localizes to mitochondria |
| MAK3 | YPR051W | Catalytic subunit of N-terminal acetyltransferase of the NatC type; required for replication of dsRNA virus |
| ATF1 | YOR377W | Alcohol acetyltransferase with potential roles in lipid and sterol metabolism; responsible for the major part of volatile acetate ester production during fermentation |
| ILV2 | YMR108W | Acetolactate synthase, catalyses the first common step in isoleucine and valine biosynthesis and is the target of several classes of inhibitors, localizes to the mitochondria; expression of the gene is under general amino acid control |
| LEU9 | YOR108W | Alpha-isopropylmalate synthase II (2-isopropylmalate synthase), catalyzes the first step in the leucine biosynthesis pathway; the minor isozyme, responsible for the residual alpha-IPMS activity detected in a leu4 null mutant |
| YPL113C | YPL113C | Putative dehydrogenase |
| AAD14 | YNL331C | Putative aryl-alcohol dehydrogenase with similarity to P. chrysosporium aryl-alcohol dehydrogenase; mutational analysis has not yet revealed a physiological role |
