# Supplementary material for: Linking gene regulation and the exo-metabolome: A comparative transcriptomics approach to identify genes that impact on the production of volatile aroma compounds in yeast
Source: BMC Genomics. 2008 Nov 7;9:530. doi: 10.1186/1471-2164-9-530 (PMC2585593; doi:10.1186/1471-2164-9-530)
Supplement: Additional file 2 — Inter- and intra-strain differences in expression levels of all identified ORFs. The three tables show the differences in expression levels of identified ORFs between different strains at day 2 (Table 1) and at day 5 (Table 2) of fermentation, and differences between day 2 and day 5 in individual strains (Table 3). [file 1471-2164-9-530-S2.doc]

**Additional data file 2**

**Table 1**

Difference in expression of identified ORFs between the five strains at day 2 of fermentation.

**Table 2**

Difference in expression of identified ORFs between the five strains at day 5 of fermentation.

**Table 3**

List of transcripts significantly up or down regulated between days 2 and 5 of fermentation in each strain.
