# Supplementary material for: Linking gene regulation and the exo-metabolome: A comparative transcriptomics approach to identify genes that impact on the production of volatile aroma compounds in yeast
Source: BMC Genomics. 2008 Nov 7;9:530. doi: 10.1186/1471-2164-9-530 (PMC2585593; doi:10.1186/1471-2164-9-530)
Supplement: Additional file 3 — Summaries of PLS1 models. The table summarises PLS1 models used for interpretation and selection of genes for overexpression. [file 1471-2164-9-530-S3.doc]

Additional data file 3

Summaries of PLS1 models used for interpretation and selection of genes for overexpression.
