# Supplementary material for: Linking gene regulation and the exo-metabolome: A comparative transcriptomics approach to identify genes that impact on the production of volatile aroma compounds in yeast
Source: BMC Genomics. 2008 Nov 7;9:530. doi: 10.1186/1471-2164-9-530 (PMC2585593; doi:10.1186/1471-2164-9-530)
Supplement: Additional file 4 — Comparison between metabolites produced in the strains overexpressing individual ORFs. The table shows a comparison between the volatile aroma metabolites that are produced in the strains overexpressing individual ORFs at day 2 and day 5 of fermentation. [file 1471-2164-9-530-S4.doc]

Additional data file 4

Comparison between metabolites produced in the strains overexpressing individual ORFs at day 2 and day 5 of fermentation
